# Correlates of the Timely Initiation of Complementary Feeding among Children Aged 6–23 Months in Rupandehi District, Nepal

**DOI:** 10.3390/children5080106

**Published:** 2018-08-06

**Authors:** Dilaram Acharya, Radha Subedi, Kwan Lee, Seok-Ju Yoo, Salila Gautam, Jitendra Kumar Singh

**Affiliations:** 1 Department of Preventive Medicine, College of Medicine, Dongguk University, Gyeongju, 123 Dongdae-ro, Gyeongju-si 38066, Korea; dilaramacharya123@gmail.com (D.A.); medhippo@hanmail.net (S.-J.Y.); 2 Department of Community Medicine, Devdaha Medical College and Research Institute, Kathmandu University, Devdaha, Rupandehi 32900, Nepal; 3 Department of Public Health, Sanjeevani College of Medical Sciences, Butwal 32907, Nepal; radhasubedi@gmail.com (R.S.); salu_but@yahoo.com (S.G.); 4 Department of Community Medicine and Public Health, Janaki Medical College, Tribhuvan University, Janakpur 45600, Nepal; jsingdj@gmail.com

**Keywords:** complementary feeding, correlates, timely initiation, Nepal

## Abstract

Although the predictors of the timely initiation of complementary feeding are well-known elsewhere, there is less awareness of the topic in Nepal. The current study was undertaken to identify the correlates of timely initiation of complementary feeding among children aged 6–23 months. A community-based cross-sectional study was conducted in the Rupandehi district, Nepal. A total of 155 mother-child pairs were selected using a simple random sampling technique. Logistic regression with adjustment for potential confounders was employed to examine the independent association between risk factors and the timely initiation of complementary feeding. Fewer than 3 in 5 children aged 6–23 months received complementary feeding at the recommended time. Literate mothers and a maternal occupation in the service or business sectors were found to be associated with complementary feeding at 6 months. In addition, child characteristics such as birth order, male children, and those fed micronutrients were also more likely to have been received complementary feeding at 6 months than their counterparts. Maternal education and occupation, and child characteristics such as, birth order, male gender, and micronutrient consumption, which are correlates of the timely initiation of complementary feeding, suggest that the Nepalese Infant and Young Child Feeding (IYCF) programme should target these predictors while designing preventive strategies.

## 1. Introduction

Malnutrition is a major public health problem. In 2017, the World Health Organization (WHO) reported that 462 million children were underweight, 52 million children below 5 years of age were low weight for their height, and 155 million children were stunted. Undernutrition contributes to about 45% of deaths among children below 5 years of age, and most deaths occur in low- and middle-income countries [1]. In Nepal, an estimated 27%, 36%, and 10% of children aged <5 years were underweight, stunted, or wasted, respectively, in 2016 [2].

Children aged 6–23 months more commonly suffer from undernutrition, since this is the period during which children change to solid foods from mother’s milk as the primary source of nutrition, and this change may contribute to problems of indigestion, infection, and/or insufficient food [3,4]. It has been recommended that breast feeding be used exclusively for 6 months, and thereafter, nutritionally safe and adequate complementary feeding in addition to breast feeding be used to meet growing nutritional needs [5,6,7]. In Nepal, among children aged 6–23 months, only 35% were fed in accordance with minimum acceptable diet criteria to 20,016 [2].

A number of studies have reported the importance of timely introduction of complementary feeding. Both early and delayed introduction of complementary feeding may lead to poor nutritional status and increased morbidity among younger children [8,9,10]. For example, a long-term deterioration in physical growth was identified in infants who received premature complementary feeding in a Vietnamese study [9], while recent systematic reviews have reported that delayed complementary feeding may increase the risk of child obesity [11,12].

A previously published paper identified that the most consistent determinants of inappropriate complementary feeding practices across Nepal, India, Bangladesh, and Sri Lanka were lack of maternal education and lower household wealth [13]. Similarly, many other studies conducted in South East Asia have highlighted levels and determinants of complementary feeding based on meal frequency [14] and changes in indicators of complementary feeding practices associated with counseling by community health workers [15]; they have developed and compared complementary feeding recommendations, evaluated the contributions of fortified foods to meet the nutrient requirements of younger children [16], and identified determinants of inappropriate complementary feeding [16,17]. However, these studies did not seek to identify factors related to the initiation of complementary feeding at recommended times.

Given the above-described background, we aimed to identify the correlates of the timely initiation of complementary feeding among children aged 6–23 months in Rupandehi district, Nepal.

## 2. Materials and Methods

### 2.1. Study Design and Setting

A community-based cross-sectional study was carried out in the Semlar Village Development Committee (VDC), which is located in a rural area of Rupandehi District, Nepal. The study area was located in the western development region of Nepal, and the VDC is the lowest administrative unit in rural areas of Nepal. At the time of the study, this VDC had a population of 10,082, and 5160 were children below the age of two years [18].

### 2.2. Sampling

Numbers of households and population in each ward of the VDC were obtained from the VDC office. Households were selected by simple random sampling. The study respondents were mothers with children 6–23 months old. We excluded children aged <6 months and >23 months. A sample size of 118 was calculated by assuming 8% of Nepalese children breastfed, and children aged 6–23 months did not receive timely the solid or semisolid complementary foods in addition to breast milk [4]; there was an allowable error 5%. The sample size was increased to 155 to account for possible missing data. 

### 2.3. Data Collection

Data were collected between 20 March 2013 and 30 April 2013 from the respondents using a structured questionnaire adapted from the Nepal Demographic and Health Survey (NDHS) 2011 questionnaire [4]. Four research assistants trained to perform interviews collected data from respondents by face-to-face interview. The questionnaire administered consisted of two parts: (i) selected baseline characteristics of participants and (ii) breast feeding and complementary feeding. The main outcome variable of this study was timely initiation of complementary feeding as recommended by WHO and UNICEF [6]. The independent study variables, which were based on categories given in NDHS 2011, were maternal characteristics (age, caste/ethnicity), type of family (as-joint or nuclear), educational status, occupation, and child characteristics (e.g., sex, birth order, breast feeding, complementary feeding initiated time, type of complementary feeding used, and micronutrient supplementation) [4].

Maternal age was categorized as 15–19 years, 20–24 years, 25–29 years, and ≥30 years. Caste/ethnicity was classified based on the caste system in Nepal and divided into three major groups based on available literature and similarities between caste and ethnic groups: (I) advantaged (Brahmin and Chetri), (II) disadvantaged (Adibasi/Janjati), and (III) disadvantaged (dalit) [19]. First birth order was categorized as ‘yes’ or ‘no’. Birth weight were categorized as: normal (≥2.5 kg) or low birth weight (<2.5 kg). Initiation of breast feeding time was coded as before one hour of birth or after one hour of birth. Continuation of breast feeding was categorized as ‘yes’ or ‘no’ (for up to at least one year). Micronutrient feeding (Baal-Vita) was categorized as ‘yes’ or ‘no’. As part of a strategy to address the growing burden of malnutrition among children, Nepal Government in collaboration with UNICEF and an implementing organization designed and launched an intervention project of an “infant and young child feeding (IYCF) and micronutrient powder (MNP) project.” The IYCF/MNP intervention included the distribution of 60 sachets of micro-nutrient powder (locally branded as “Baal Vita,” which contain multivitamins and minerals—Table 1) through local health facilities or Female Community Health Volunteers (FCHVs) to all children aged 6–23 months of age every six months with the suggestion to feed the child one sachet of Baal-Vita every day mixed into food for two months. Every six months, the families were requested to come back and pick up a new batch of 60 sachets so that the child should consume 180 sachets over the eligible period of 18 months [20,21,22].

### 2.4. Ethical Considerations

The study protocol was approved by the Ethical Committee for Health Research of Sanjeevani College of Medical College Sciences, Purbanchal University, Nepal (Ref. 45-20-03-2013). Approval letters were also obtained from District Public Health Office, Rupandehi, and Semlar Village Development Committee. Mothers provided written consent before interview, and all personal identifiers were removed before data analysis. 

### 2.5. Statistical Analyses

Demographic characteristics, health related characteristics, and complementary feeding of children were presented in frequency and percentage. Timely initiation of complementary feeding (at 6 months) was the main variable of interest that was dichotomized into yes (*n* = 89) and no (*n* = 66). The association between independent variables and timely initiation of complementary feeding was examined using chi-square (χ^2^) test. Then, significant variables based on chi-square results with *p*-value less than 0.05 were analyzed using multivariate logistic regression. Odds ratio (OR) with 95% confidence interval (CI) were obtained to ascertain the association between explanatory and dependent variables, and a *p* value of less than 0.05 was considered as statistically significant.

## 3. Results

### 3.1. Association between Maternal Socio-Demographic Characteristics, Health-Related Characteristics, and Complementary Feeding Practices in Children Aged 6–23 Months

Baseline maternal and child characteristics and complementary feeding practices are summarized in Table 2. More than half of the mothers (56.8%) were aged from 15 to 24 years old, joint families (70.3%), of Brahmin or Chhetri ethnicities (53.5%), Hindus (94.4%), and housewives (90.3%). Slightly more than half (57.4%) of the children were in first birth order, females (52.3%), aged 6–12 months (66.5%), normal birth weight (80.6%), breast-fed within first hour of birth (58.5%), still breast feeding continued (91.6%), micronutrient fed to the baby (55.5%), complementary feeding at 6 months (57.4%), and majority (60%) achieved minimum dietary diversity (MDD). Association between maternal socio-demographic characteristics, health-related characteristics, and timing of complementary feeding by univariate analysis has demonstrated that maternal characteristics such as education and occupation, and health-related characteristics such as birth order of children, sex, birth weight, and children who were fed with micronutrients, were significantly associated with receiving complementary feeding at 6 months (*p* < 0.05).

### 3.2. Association of Maternal Socio-Demographic Factors, Health-Related Characteristics, and Complementary Feeding at 6 Months in Children Aged 6–23 Months

Association between complementary feeding received by children at 6 months and maternal socio-demographic factors; health-related characteristics are summarized in Table 3. A number of health related maternal and child factors, that is, educational status and occupation of mother; birth order, sex, and micronutrient feeding, were significantly associated with receiving complementary feeding at 6 months by univariate analysis (Table 3). Multivariate analysis adjusted for co-variates showed that educational status and occupation of mother, birth order, sex, birth weight, and micronutrient feeding were significantly associated with receiving complementary feeding at 6 months (Table 3). 

## 4. Discussion

Reports previously published in many developing countries, including Nepal, have sought to investigate the determinants of inappropriate complementary feeding [23,24,25,26,27,28]. In the present study, we explored some of the specific gaps in meeting the timely initiation of complementary feeding among children aged 6–23 months as per the newly established criteria recommended by the WHO. We found slightly more than half (57.4%) received complementary feeding at 6 months, which is lower than the national average as determined by the Nepal Demographic and Population Health Survey 2016 [2]. NDHS 2016 reported eighty-three percent of children aged 6–8 months receive timely complementary foods, and only 10% of children aged 18–23 months were weaned. However, this finding of our study is similar to some recently published papers [29,30]. Study from Ethiopia [30] reported that 61% of children aged 6–23 months received complementary feeding, while another review paper reported 57.4% of South Asian children of same age group were introduced to timely complementary feeding [29]. However, studies from Bangladesh and southern Ethiopian studies stated nearly three fourth of children initiated timely complementary feeding [25,31]. Differences might be due to the variation in the attributes of study samples, in addition to other inherent explanatory characteristics such as education, socio-economic condition, and use of maternity care services [32,33,34]. Educational level, occupation of mother, parity, having ANC follow up, and birth preparedness have been reported to be independent predictors of timely initiation of complementary feeding [33].

In the present study, we found that maternal personal characteristics, such as educational status and occupation, were significantly associated with the timely initiation of complementary feeding to children aged 6–23 months. Children of mothers that were literate and engaged in income generating activities were found to be more likely to receive timely complementary feeding. Studies conducted in Bangladesh, India, and Ethiopia have also reported a similar association [25,33,35]. In addition, study from seven African countries also reported that poor households and non-working mothers were the main factors associated with suboptimal complementary feeding [27]. In the line of our study finding, Patel A. et al. also stated mothers from higher income households were more likely to introduce timely complementary foods to their children [23]. Employed educated women are probably more aware of the importance of initiating timely complementary feeding to their children. These findings indicate that the IYCF programme of Nepal should target illiterate mothers and housewives, and that, in the long-term, non-formal education and introduction to income generating activities should be recommended to this population to increase the proportion of children afforded complementary feeding in a timely manner. 

Interestingly, we identified children with birth order of more than one, and male genders were also more likely to have been received complementary feeding at 6 months. Child characteristics, such as birth order [35] and male gender [36], have been reliably reported to be determinants of the timely initiation of complementary feeding. Increased awareness might have developed among mothers with a number of births that might lead to timely initiation of complementary feeding to their children. Similarly, it might be possible; the traditional gender norm [36] could have been influenced by timely complementary feeding of their male children. Moreover, our study revealed that those children who were fed with micronutrients more commonly received timely complementary feeding. This could be the influential role of micronutrient supplementation programme [37]; furthermore, IYCF/MNP intervention programme of Nepal [21] includes the provision of behavior change information, counseling, and support regarding recommended IYCF practices. Micronutrient supplementation program in Nepal is associated with nutrition education and counseling. Thus, increased distribution of micronutrients sachets might increase the percentage of timely initiation of complementary feeding.

Our study has some important strengths. First, we enrolled the study participants from different socio-economic conditions representative of district and region of the country. Second, findings of the study can be used to inform various stakeholders and policy makers in drafting future intervention programmes to improve complementary feeding practices among Nepalese infants and young children. However, in spite of our efforts, this study has some limitations. Firstly, the sample size is relatively small; however, the results from the representative random sample could be generalized at least to similar settings. Secondly, the study assessed only correlates of the timely initiation of complementary feeding among children aged 6 to 23 months, and other important complementary feeding indicators, such as minimum dietary diversity, minimum meal frequency, and minimum acceptable diet were not investigated. Further, studies of the impact of timely initiation of complementary feeding on child health outcome are recommended. 

## 5. Conclusions

Less than three in five mothers were found to initiate complementary feeding when children were sixth months old. Maternal education and occupation and child characteristics, such as birth order, sex, and micronutrient consumption, were determined to be correlates of the timely initiation of complementary feeding. The IYCF programme should target these predictors while designing preventive strategies. Lastly, studies on the impact of timely initiation complementary feeding on child health are recommended. 

## Figures and Tables

**Table 1:** Contents of Micronutrient Powder—Baal-vita (in One Gram Sachet).

Micronutrient	Amount	Micronutrient	Amount
Vitamin A	400 μg	Vitamin B12	0.5 mg
Vitamin C	60.0 mg	Folic acid	150 μg
Vitamin D	5.0 μg	Iron	10.0 mg
Vitamin E	5.0 mg	Zinc	4.1 mg
Vitamin B1	0.5 mg	Copper	0.56 mg
Vitamin B2	0.5 mg	Selenium	17.0 mg
Niacin	6.0 mg	Iodine	90.0 μg
Vitamin B6	0.9 μg	Vitamin B12	0.5 mg

**Table 2:** Association between maternal socio-demographic characteristics, health-related characteristics, and complementary feeding practices in children aged 6–23 months (*n* = 155) *.

Variables	*n* = 155 (%)	Complementary Feeding at 6 Months		*p* value
		Yes *n* = 89 (%)	No *n* = 66 (%)	
Age of mother				0.827
15–19 years	13 (8.4)	6 (46.2)	7 (53.8)	
20–24 years	75 (48.4)	45 (60.0)	30 (40.0)	
25–29 years	60 (38.7)	34 (56.7)	26 (43.3)	
≥30 years	7 (4.5)	4 (57.1)	3 (42.9)	
Types of Family				0.615
Joint	109 (70.3)	64 (55.6)	45 (44.4)	
Nuclear	46 (29.7)	25 (57.5)	21 (42.5)	
Ethnicity				0.203
Dalit	16 (10.3)	6 (37.5)	10 (62.5)	
Adibasi/Janajati	56 (36.1)	35 (62.5)	21 (37.5)	
Brahmin/Chhetri	83 (53.5)	48 (57.8)	35 (42.2)	
Religion				0.907
Hindu	146 (94.4)	84 (57.5	62 (42.5)	
Buddhist	9 (5.6)	5 (55.6)	4 (44.4)	
Educational status				0.033
Illiterate	28 (18.1)	10 (35.7)	18 (64.3)	
Primary	22 (14.2)	11 (50.0)	11 (50.0)	
Secondary	63 (40.6)	39 (61.9)	24 (38.1)	
SLC and above	42 (27.1)	29 (69.0)	13 (31.0)	
Occupation				0.016
Service/business	15 (9.7)	13 (86.7)	2 (13.3)	
Housewife	140 (90.3)	76 (54.3)	64 (45.7)	
First baby in the family				0.020
No	66 (42.6)	45 (68.2)	21 (31.8)	
Yes	89 (57.4)	44 (49.4)	45 (50.6)	
Sex of baby				0.0001
Male	74 (47.7)	58 (78.4)	16 (21.6)	
Female	81 (52.3)	31 (38.3)	50 (61.7)	
Age of children				0.775
6–12 months	52 (33.5)	26 (50.0)	26 (50.0)	
12–23 months	103 (66.5)	49 (47.6)	54 (52.4)	
Birth weight of baby				0.032
Normal (≥2.5 kg)	125 (80.6)	77 (61.6)	48 (38.4)	
Low birth weight (<2.5 kg)	30 (19.4)	12 (40.0)	18 (60.0)	
Initiation of breast feeding ^a^				0.156
After one hour	59 (41.5)	39 (66.1)	20 (33.9)	
Within one hour	83 (58.5)	45 (54.2)	38 (45.8)	
Still breast feeding continued				0.754
No	13 (8.4)	8 (61.5)	5 (38.5)	
Yes	142 (91.6)	81 (57.0)	61 (43.0)	
Micronutrient fed to baby				0.037
Yes	69 (44.5)	46 (66.7)	23 (33.3)	
No	86 (55.5)	43 (50.0)	43 (50.0)	
Minimum dietary diversity (MDD)				0.512
Yes	93 (60)	50 (53.8)	43 (46.2)	
No	62 (40)	30 (48.4)	32 (51.6)	

* Chi-square (χ^2^) test was employed, and a *p*-value < 0.05 was considered as statistically significant. ^a^ missing = 13.

**Table 3:** Association of maternal socio-demographic factors, health-related characteristics, and complementary feeding at 6 months in children aged 6–23 months as determined by logistic regression analysis ^a^.

Unadjusted Analysis			
**Factors**	**Category**	**OR**	**95% CI**
Age of mother	20 years & more vs. less than 20	1.6	0.5–5.1
Types of family	Joint vs. Nuclear	1.1	0.6–2.3
Ethnicity	Dalit vs. Brahmin/Chhetri	0.3	0.1–1.1
	Janajati vs. Brahmin/Chhetri	0.4	0.1–1.3
Religion	Hindu vs. Buddhist	1.0	0.2–4.2
Educational status	Literate vs. Illiterate	3.9	1.7–9.0
Occupation	Service/Business vs. Housewife	5.4	1.1–25.1
First baby in the family	No vs. Yes	2.1	1.1–4.2
Sex of baby	Male vs. Female	5.8	2.8–11.9
Birth weight of baby	Normal vs. LBW	2.4	1.0–5.4
Initiation of breast feeding	Within 1 h vs. After 1 h	0.6	0.3–1.2
Still breast feeding continued	Yes vs. No	0.8	0.2–2.6
Micronutrient fed to baby	Yes vs. No	2.0	1.0–3.8
**Factors**	**Category**	**AOR**	**95% CI**
Educational status	Literate vs. Illiterate	2.9	1.1–8.2
Occupation	Service/Business vs. Housewife	13.1	2.1–82.2
First baby in the family	No vs. Yes	3.9	1.6–9.3
Sex of baby	Male vs. Female	6.3	2.7–14.5
Micronutrient fed to baby	Yes vs. No	2.5	1.1–5.7

^a^ Variable entered: educational status, occupation, first baby in the family, sex of baby, birth weight of baby, and micronutrient fed to baby.
